# Decoding Immunotherapy Response in Colorectal Cancer: Translational Insights Beyond MSI

**DOI:** 10.3390/cancers18050852

**Published:** 2026-03-06

**Authors:** Chiara Cataldi, Beliz Bahar Karaoğlan, Elena Liotta, Sara De Dosso

**Affiliations:** 1 Department of Molecular Medicine, Sapienza University of Rome, 00161 Rome, Italy; chiara.cataldi@uniroma1.it; 2 Eskişehir City Hospital, 26080 Eskişehir, Turkey; bbaharulas@gmail.com; 3 Department of Radiological, Oncological and Pathological Science, Sapienza University of Rome, 00161 Rome, Italy; elena.liotta@uniroma1.it; 4 Medical Oncology Department, Oncology Institute of Southern Switzerland (IOSI), Ente Ospedaliero Cantonale (EOC), 6500 Bellinzona, Switzerland; 5 Faculty of Biomedical Sciences, University of Southern Switzerland (USI), 6900 Lugano, Switzerland

**Keywords:** colorectal cancer, immunotherapy, biomarkers

## Abstract

**Background/Objectives**: Immune checkpoint inhibitors (ICIs) are among the transformative and manageable systemic therapies for several cancer types, including colorectal cancer (CRC). Nevertheless, their clinical benefit is limited to mismatch-deficient or microsatellite instability-high diseases, which represent only a small percentage of cases. Despite this initial major and stringent selection, primary and acquired resistance remain clinically relevant. Therefore, the identification of additional biomarkers is essential to refine patient selection and guide rational combinational strategies. This review aims to summarize the current evidence regarding established and emerging biomarkers of response and resistance to ICIs in CRC. **Methods**: This narrative review identified and synthesized relevant clinical trials, translational studies, and reviews through a literature search of emerging biomarkers of immunotherapy response in colorectal cancer. **Results**: Deficient mismatch repair/high microsatellite instability remains the most reliable predictive biomarker of ICI response, emphasized by high tumor mutational burden, *POLE*/*POLD* mutations, and specific tumor microenvironment features. Emerging indicators, including molecular alterations, antigen presentation machinery integrity, PD-L1-mediated signaling, microbiome connections, and circulating tumor DNA kinetics, have demonstrated significant potential as sources for therapeutic response prediction and have informed the development of innovative combination strategies in both MSI-H and MSS CRCs. **Conclusions**: Immunotherapy response in CRC is determined by a complex interplay between tumor-intrinsic, immune, microenvironmental, and systemic factors. Integrating multiple biomarkers may provide superior stratification and guide therapeutic strategies. Prospective validation and standardized biomarker assessment will be imperative to translate these insights into clinical practice.

## Simple Summary

Immunotherapy is redefining the clinical management of colorectal cancer. However, its broader application remains limited to a well-defined subgroup of patients. As an emerging therapeutic strategy in this context, further investigation is warranted to fully realize its therapeutic potential. This review aims to provide a comprehensive overview of biomarkers for immune checkpoint inhibitors, evolving beyond the established mismatch repair status to refine patient selection, maximize clinical benefits, minimize toxicities, and circumvent the initiation of suboptimal therapies.

## 1. Introduction

Colorectal cancer (CRC) remains one of the most diagnosed cancers worldwide and a leading cause of cancer-related morbidity and mortality. Approximately 20% of patients are diagnosed with metastatic disease, associated with limited long-term survival despite modern systemic therapies [1]. Recently, immunotherapy has transformed the therapeutic paradigm for many solid tumors. However, its therapeutic success in CRC is limited to a biologically distinct subgroup characterized by deficient mismatch repair (dMMR) or high microsatellite instability (MSI-H). The prevalence of mCRC is approximately 15% of all CRCs and 3–5% of metastatic cases [2]. This disparity reflects a distinct biological behavior: MSI-high/MMR-deficient colorectal cancers typically remain locally confined, exhibiting prominent local growth and invasion rather than early distant dissemination [3].

Pivotal trials, such as KEYNOTE-177, CheckMate-142, and more recently, CheckMate-8HW, have demonstrated significant and durable responses in these patients, establishing immune checkpoint inhibitors (ICIs) as the standard of care in the first-line setting [4,5,6]. Similarly, neoadjuvant ICIs have provided unprecedented pathological complete responses, exceeding 60–70% in early-stage CRC, facilitating non-operative management in selected patients [7,8,9]. Collectively, these results suggest that immunotherapy induces deep responses as well as possesses the potential to fundamentally alter disease progression.

Despite these encouraging advances, not all dMMR/MSI-H tumors respond to ICIs. A subset of patients exhibits primary resistance, defined as the absence of meaningful tumor regression or early disease progression despite treatment initiation. Others experience secondary (acquired) resistance, characterized by disease progression following an initial clinical response or disease stabilization. Primary resistance may reflect intrinsic tumor features—such as impaired antigen presentation, alternative immune-evasion pathways, or a non-inflamed tumor microenvironment—whereas secondary resistance likely results from tumor evolution under therapeutic pressure. This includes neoantigen loss, alterations in interferon signaling, or adaptive changes in the tumor microenvironment. These observations underscore that MSI-H/dMMR status alone does not fully capture the complexity of immunotherapy responsiveness, highlighting the need for additional predictive biomarkers to refine patient selection and improve clinical outcomes.

Conversely, the remaining larger group of CRCs (approximately 97–95% of metastatic cases), characterized by microsatellite-stable (MSS) disease, derives marginal clinical benefit from ICIs, primarily due to the immune-excluded microenvironment [10,11,12].

Given these clinical challenges, canonical MMR/MSI status is insufficient for complete patient selection. Consequently, a deeper understanding of tumor-intrinsic, immunologic, microenvironmental, genomic, and circulating biomarkers is essential to predict ICI outcomes, primary and acquired resistances, and their mechanisms, and to guide rational combination strategies to improve ICI benefits in CRCs. This review aims to report established and emerging biomarkers of ICI efficacy and resistance in CRCs, providing an integrated framework to enhance patient stratification and personalized therapeutic strategies.

## 2. Canonical Biomarkers

In CRC, patient selection for ICIs is contingent upon a limited set of clinically established and extensively validated biological features that guide therapeutic decision-making in routine clinical practice.

Microsatellite instability is the most validated biomarker for immunotherapy in CRC. It results from the loss of function in mismatch repair genes (*MLH1*, *MSH2*, *MSH6*, and *PMS2*). This impairment, termed deficient MMR (dMMR), leads to the accumulation of mutations in short, repetitive DNA sequences called microsatellites, resulting in the MSI-H phenotype. Although used interchangeably, dMMR describes the functional defect, while MSI-H refers to its molecular consequence. MSI status is routinely assessed by immunohistochemistry (IHC), polymerase chain reaction assays (PCR), or next-generation sequencing (NGS) [13,14]. In the tumor microenvironment (TME), MSI-H CRCs are associated with high tumor mutation burden and increased neoantigen expression, which stimulates immune response by promoting CD8+ T-cell infiltration, increased PD-L1 expression, and antitumor activity [15,16]. In contrast, microsatellite-stable (MSS) CRCs typically demonstrate suboptimal responses to ICIs, largely due to their non-immunological TME. Nevertheless, ongoing clinical studies are exploring strategies to mitigate this resistance in MSS CRC, including combination therapies aimed at modifying the TME and enhancing immune infiltration [12,17,18,19].

Beyond MSI/dMMR status, tumor mutational burden (TMB) is considered a measure of tumor immunogenicity. TMB corresponds to the total number of somatic coding mutations per megabase (mut/Mb) of the tumor genome and is typically measured using NGS methods [20]. In various cancer types, elevated TMB correlates with good responses to ICIs, increased neoantigen formation, and tumor immunogenicity. Pembrolizumab was approved by the FDA for patients with metastatic solid tumors and TMB ≥ 10 mut/Mb, independent of tumor histology, based on the phase II KEYNOTE-158 trial. However, CRC was notably underrepresented in this trial [21]. In the TAPUR basket study, patients with TMB-H mCRC demonstrated a modest disease control rate of 31% when treated with pembrolizumab [22]. Nivolumab plus ipilimumab also yielded suboptimal results [23]. These findings demonstrate that a universal cutoff of ≥10 mut/Mb may be insufficient to predict benefit in CRC. Additionally, although MSI-H tumors are typically characterized by elevated TMB, the two parameters are not interchangeable, as high TMB in CRC may arise from distinct biological mechanisms—such as polymerase proofreading defects—and does not uniformly translate into immune sensitivity. Furthermore, TMB is not currently recommended as a standalone biomarker outside of clinical trials, and the NCCN guidelines suggest a higher threshold of 50 mut/Mb for CRC.

Within this established framework, aberrations in DNA replication fidelity also serve as another source of hypermutation with relevant implications for tumor immunogenicity and response to ICIs. The proofreading domains of DNA polymerases ε (POLE) and δ (POLD) typically correct errors during DNA replication. Pathogenic variants in these genes reduce the accuracy of proofreading, leading to somatic mutations and producing an ultra-hypermutated phenotype, which is typically characterized by TMB-H tumors [24]. Colorectal carcinogenesis can be predisposed by somatic mutations as well as germline variants linked to polymerase proofreading-associated polyposis (PPAP). The ultra-hypermutated state enhances tumor immunogenicity, resulting in dense immune infiltration and heightened sensitivity to ICIs [25]. Compared to patients with non-pathogenic *POLE*/*POLD* variants, retrospective analyses have demonstrated that patients with pathogenic variants benefit significantly from PD-1 blockade, with overall response rates (ORRs) exceeding 80%, combined with prolonged survival times. Even in MSS backgrounds, recent clinical reports corroborate durable responses in mCRC [26,27]. Considering this data, the NCCN (National Comprehensive Cancer Network) guidelines now advise treating mCRC with pathogenic *POLE*/*POLD* variants in accordance with the same guidelines as MSI-H/dMMR tumors.

However, within the European regulatory context, TMB or *POLE*/*POLD* pathogenic variants do not currently represent approved indications for immune checkpoint inhibition. Consequently, despite NCCN recommendations and compelling translational data, their use in Europe remains restricted to off-label settings or clinical trials. This underscores a gap between biological evidence, clinical guidelines, and regulatory approval. This regulatory discrepancy carries significant ethical and clinical implications. European patients harboring POLE/POLD1 pathogenic variants or high TMB often face limited access to immune checkpoint inhibitors outside clinical trials, despite biologically compelling evidence and international guideline support. Consequently, inequities in access to precision oncology arise between geographical regions. From a research perspective, the absence of regulatory approval hinders the accumulation of real-world data in these molecularly defined subgroups, as off-label use is restricted, and trial availability remains limited. Simultaneously, balancing early access to potentially effective therapies with the need for robust, prospective evidence requires careful consideration.

Combined with tumor genomic features, the TME plays a central role in modulating immune activation and, therefore, ICI responses. The TME is a composite ecosystem in which tumor, stromal, and immune cells dynamically interact, influencing disease courses and therapeutic responses. CRC cells alter TME composition to evade the immune system. Immune-escape mechanisms include impaired antigen presentation, recruitment of regulatory T cells (Tregs) and myeloid-derived suppressor cells (MDSCs), and macrophage polarization toward the tumor-promoting M2 phenotype. Ultimately, this results in the compromise of protective CD8+ T-cell-mediated immunity and the promotion of an immunosuppressive niche, particularly in MSS tumors [28,29,30,31]. In contrast, immune-inflamed TMEs possessing high-density tumor-infiltrating lymphocytes (TILs), especially CD8+ T cells, tertiary lymphoid structures, and interferon-γ-driven signaling are consistently associated with enhanced sensitivity to ICIs [32]. In MSI-H/dMMR mCRC, increased TIL density has been correlated with improved response rates and survival outcomes under ICIs, reinforcing its predictive value [33]. Transcriptomic analyses performed on samples from different MSI mCRC patients treated with ICIs, stratified for TME composition and cell proliferative activity, have further highlighted that TMEs possessing dense stromal fibroblast-derived components, coupled with low proliferative activity, correlated with worse outcomes. This suggests that stromal dominance may drive immune exclusion and suboptimal prognosis even in MSI tumors [11]. In parallel, metabolic and physicochemical alterations, including arginine depletion, lactate accumulation, hypoxia, and ammonia accumulation, reprogram immune cell metabolism, induce T-cell exhaustion, and further limit the antitumor efficacy of ICIs [34,35,36]. Table 1 summarizes canonical biomarkers of response to ICIs in CRC.

## 3. Coexisting Molecular Alterations

### 3.1. BRAF V600E 

The *BRAF* V600E mutation occurs in approximately 8–12% of CRCs, often coexisting with MSI, CIMP phenotype, and CMS1 immune signatures [37]. While it portends suboptimal prognosis in MSS tumors, its predictive value for immune checkpoint inhibition remains controversial. Early retrospective data suggested worse outcomes in dMMR/MSI-H mCRC, with lower 1- and 2-year progression-free survival (PFS) in *BRAF*-mutated versus wild-type tumors [38]. However, prospective trials such as KEYNOTE-177 and CheckMate-8HW did not consistently confirm suboptimal outcomes of PD-(L)1 blockade in this subgroup [4,6].

Recent real-world analyses have further clarified the role of *BRAF* V600E in immune checkpoint inhibition. In a large international cohort of 909 patients with MSI-H metastatic colorectal cancer treated with ICIs, BRAF mutation was not independently associated with inferior progression-free or overall survival [39]. However, subgroup analyses suggested a more nuanced pattern: in the first-line setting, patients receiving PD-1 monotherapy exhibited higher rates of secondary resistance compared with those treated with combined PD-1 and CTLA-4 blockade, indicating that dual checkpoint inhibition may partially overcome adaptive resistance mechanisms in this subgroup. Similar observations were reported in an independent cohort, where BRAF-mutated patients experienced less favorable outcomes under PD-(L)1 monotherapy, while the addition of CTLA-4 inhibition appeared to mitigate this difference [40]. Collectively, these findings suggest that BRAF V600E is not a uniform negative prognostic factor under ICI therapy but may influence patterns of resistance depending on treatment strategy.

Following ICI failure, BRAF/EGFR ± MEK inhibition achieved comparable activity in both dMMR and pMMR tumors, indicating retained therapeutic potential beyond immunotherapy [41]. Mechanistically, oncogenic BRAF/MAPK signaling upregulates PD-L1 expression via c-JUN/YAP transcriptional activation, linking pathway hyperactivation to immune evasion and potential PD-1 sensitivity [42]. Translational analyses from a phase II trial combining PD-1, BRAF, and MEK inhibition in *BRAF* V600E CRCs demonstrated heightened immune gene expression and enhanced MAPK suppression in responders, substantiating the synergy between targeted and immune blockade [43,44]. Likewise, early clinical evidence from SWOG-2107 suggests that encorafenib, cetuximab, and nivolumab may achieve clinically meaningful responses in *BRAF*-mutant MSS CRC, reinforcing this hypothesis [44,45].

Complementary preclinical work indicates that combined BRAF/MEK inhibition can restore antigen-presenting machinery (APM) and activate cytotoxic T cells, enhancing ICI efficacy in *BRAF*-mutant CRC [46]. Collectively, current evidence suggests that while *BRAF* V600E is not a universal negative predictor of ICI response in dMMR/MSI-H mCRC, it may favor secondary resistance under PD-1 monotherapy. Dual checkpoint blockade or targeted immune combinations represent potential avenues to circumvent this adaptive resistance.

### 3.2. KRAS

*KRAS* represents one of the most common oncogenic driver mutations in CRC, with mutations occurring in approximately 40% of cases. The main effects of these mutations result in the constitutive activation of the KRAS protein, which persistently activates downstream signaling pathways driving cell proliferation, survival, and tumorigenesis [47]. Due to these biological implications, *KRAS* mutations are usually tested in clinical practice to guide treatment decisions. Historically considered “undruggable,” the recent development of allele-specific inhibitors, such as *KRAS* G12C-targeting agents, is renewing therapeutic interest in this subgroup.

Regarding immunotherapy, *KRAS* mutational status has been demonstrated to influence clinical outcomes in MSI-H/dMMR CRC. In the CheckMate-142 trial, nivolumab plus low-dose ipilimumab induced durable responses with 24-month PFS and OS rates of 74% and 79%, respectively, with clinical benefit observed irrespective of *KRAS* or *BRAF* mutation status [5]. Similar results were found in CheckMate-8HW, as well as in the neoadjuvant setting of the NICHE-2 trial, indicating that *KRAS* does not stratify response to ICI combinations in these settings [6,7]. Notably, in KEYNOTE-177, the PFS benefit of pembrolizumab over chemotherapy was attenuated in the *KRAS*-mutant-type subgroup (HR 0.88). However, these exploratory findings should be interpreted with caution, given the limited sample size and the consequent lack of statistical power for subgroup comparisons [4].

Nevertheless, recent preclinical and translational evidence suggest a broader immunomodulatory role of *KRAS*. Its mutations can influence the TME by promoting the secretion of anti-inflammatory cytokines (IL-10, TGF-β, GM-CSF), downregulating MHC class I expression, and upregulating PD-L1 proliferative signaling. The main result is reduced cytotoxic CD8^+^ T-cell activity and promotion of immune evasion [48]. Conversely, *KRAS* may also induce pro-inflammatory mediators, including ICAM-1 or IL-8, contributing to a pro-tumoral TME [49]. Other mechanistic studies have identified novel pathways of *KRAS*-mediated immune escape: *KRAS* G12D represses IRF2, leading to CXCL3 overexpression and recruitment of MDSCs via CXCR2, which confers primary resistance to anti-PD-1 therapy. Conversely, restoring IRF2 expression or inhibiting CXCR2 resensitizes tumors to ICI in murine models [50]. Importantly, early-phase clinical trials targeting the CXCR2 axis are currently underway in advanced solid tumors, including colorectal cancer. For example, navarixin (MK-7123) combined with pembrolizumab was investigated in a phase II study involving patients with microsatellite-stable (MSS) colorectal cancer (NCT03473925) [51,52]. While the combination demonstrated a manageable safety profile, objective responses in the MSS CRC cohort were limited, highlighting the biological complexity of overcoming immune resistance in this setting. Similarly, the phase I/II STOPTRAFFIC-1 trial (NCT04599140) is evaluating the dual CXCR1/2 inhibitor SX-682 in combination with nivolumab in patients with RAS-mutated, MSS metastatic colorectal cancer. This study aims to determine safety, optimal dosing, and preliminary efficacy, while also exploring pharmacodynamic effects on tumor-associated neutrophils and myeloid-derived suppressor cells. Although definitive efficacy results are awaited, such trials represent an important effort to translate CXCR2-targeting strategies into immune-refractory colorectal cancer.

Finally, targeting the *KRAS*-regulated metabolic transporter SLC25A22 reduces MDSC infiltration, increases CD8^+^ T cells, and synergizes with PD-1 blockade in *KRAS*-mutant CRC models [53].

In addition, transcriptomic analyses demonstrated increased *KRAS*-related signatures, in conjunction with EMT, angiogenesis, and TGF-b signaling, in non-responders to PD-1 blockade in MSI-H/dMMR gastrointestinal tumors [54]. Collectively, these findings substantiate the role of KRAS pathway activation as a potential driver of immune exclusion and resistance, providing a rationale for combinational strategies of ICIs with the targeted inhibition of KRAS downstream effectors in CRC. Several early clinical trials are currently ongoing. Notably, a multicenter phase 1b/2 trial (NCT06412198) is investigating the combination of adagrasib, cetuximab, and the anti-PD-1 antibody cemiplimab in patients with *KRAS* G12C-mutated, pretreated mCRC. This trial reflects a biologically driven strategy designed to reverse immune resistance by simultaneously targeting oncogenic signaling, adaptive feedback through *EGFR*, and immune exhaustion, although clinical efficacy data are currently pending.

### 3.3. Emerging Molecular Signatures

*HER2* (*ERBB2*) amplification or overexpression occurs in approximately 3–5% of pMMR/MSS, *RAS*/*BRAF* wild-type CRCs. It has recently emerged as an actionable oncogenic driver [55,56]. In a large pooled analysis of patients with untreated mCRC, HER2-positive tumors displayed shorter PFS and OS, confirming its negative prognostic value and lack of predictive interaction with anti-*EGFR* or bevacizumab benefit [57]. Recent translational studies suggest that *HER2* amplification contributes to shaping a non-immunogenic TME, with functionally impaired cytotoxic infiltration and reduced interferon signaling, which may suggest limited responsiveness to immune checkpoint blockade [56,58,59]. Moreover, *HER2* mutations, distinct from gene amplification, are enriched in MSI-H CRC and correlate with inferior PFS during PD-1 blockade [60], substantiating its potential role as a biomarker of immune resistance. While HER2–PD-1 combination strategies have demonstrated benefit in gastric cancer, their role in CRC remains exploratory, warranting further translational investigation [61].

Recent evidence has renewed interest in the PI3K pathway in CRC. The PI3K/AKT/mTOR axis plays a crucial role in CRC by regulating cell growth, metastasis, and therapy resistance [62]. *PI3KCA*-mutated tumors have been associated with higher TMB, which may suggest potential immunogenicity [63]. However, no direct clinical evidence currently links *PI3K* alterations (observed in 20–25% of CRC) to ICI response in CRC. Preclinical data substantiate the role of *PI3K* activation in immune evasion: in a syngeneic MC38 model, expression of the oncogenic *PI3KCA H1047* mutation reduced CD8^+^ T-cell infiltration, increased suppressive myeloid cells, and conferred resistance to PD-1 blockade. This resistance was reversed by pharmacological PI3K inhibition or *Ccl2* suppression [64]. Clinically, *PTEN* mutations—functionally leading to PI3K pathway activation—have been associated with lower ORRs, shorter PFS, and an immunosuppressive microenvironment characterized by decreased CD8^+^ T cells and macrophage enrichment in MSI-H/dMMR gastrointestinal tumors [65]. Beyond CRC, additional preclinical evidence demonstrated that the pan-PI3K inhibitor KTC1101 significantly enhanced anti-PD-1 efficacy across multiple cell lines and murine models [66]. Translational analyses from the MARIO-3 trial confirmed that the PI3K-g inhibitor eganelisib reprograms tumor-associated macrophages (TAMs) and induces immune activation, substantiating its combination with ICIs and chemotherapy to counteract TAM-driven ICIs resistance [67]. Collectively, these data suggest that PI3K pathway activation may contribute to immune resistance, and dual PI3K and PD-(L)1 blockade may prove to be a potential combinatorial strategy in CRC.

*NTRK* gene fusions are very uncommon in CRCs, occurring in <1% of unselected cases, but are more frequently observed in MSI-H tumors, particularly *BRAF* WT cases [68,69]. This distinct molecular subgroup is characterized by elevated TMB and increased neoantigen load, suggesting a potential sensitivity to immune checkpoint blockade [70,71]. Screening studies revealed that *NTRK* fusions predominantly occur in right-sided, *MLH1/PMS2*-deficient, *BRAF* wild-type CRCs, where pan-Trk IHC demonstrated high specificity for detection [72]. Moreover, molecular profiling indicated that *NTRK*-driven CRCs demonstrate mutual exclusivity with *RAS*/*BRAF* mutations but coexistence with *POLE/POLD1* aberrations substantiating a hypermutated, immunogenic phenotype [70]. Recent transcriptomic analyses indicate that MSI-H CRCs with targetable alterations such as *NTRK* or *FGFR2* mutations exhibit an immunologically active microenvironment, with higher interferon-g and cytotoxic gene signatures and reduced epithelial-mesenchymal transition pathways [71]. Collectively, these findings suggest that *NTRK* fusions may predict response to TRK-targeted therapies as well as mark a subset of MSI-H tumors with heightened immunogenic potential, which warrants further exploration in ICI trials.

*STK11*/*LKB1* is a serine/threonine kinase and tumor suppressor that regulates cell growth, metabolism, and polarity. Germline mutations cause Peutz–Jeghers syndrome, while sporadic mutations are prevalent across many solid tumors, revealing emerging therapeutic vulnerabilities [73]. In non-small-cell lung cancer (NSCLC), *STK11* mutations correlate with poor prognosis and resistance to ICIs, substantiating their potential as negative predictive biomarkers [74]. In CRC, evidence remains limited. A recent exploratory study found that reduced *STK11* expression correlated with reduced survival outcomes, particularly in *KRAS* mutant tumors. However, it did not indicate an association with immune infiltration, highlighting the need for further validation in larger, prospective cohorts [75].

All these molecular alterations are summarized in Table 2.

Preclinical studies have demonstrated that *β-catenin* activation, driven by CTNNB1 mutation, impairs dendritic cell recruitment by suppressing CCL4 expression, thereby limiting CD8^+^ T-cell priming and promoting a non-T-cell-inflamed tumor microenvironment [76]. Subsequent analyses across solid tumors confirmed that WNT/β-catenin pathway activation correlates with immune exclusion and reduced interferon-γ signatures [77]. In colorectal cancer, transcriptomic and biomarker analyses have similarly associated WNT pathway activation and related molecular signatures with reduced immune infiltration and heterogeneous responses to immunotherapy, even within MSI-H/dMMR cohorts [78,79]. However, direct clinical evidence linking CTNNB1 mutation status *per se* to ICI outcomes remains limited.

## 4. Immunological Background

### 4.1. Antigen-Presenting Machinery

The APM is the core of signal 1 of the cancer-immune cycle. The proteasome digests antigens into peptides, which are translocated to the endoplasmic reticulum (ER) by TAP1/2, digested by ERAP1/2, and loaded into the HLA class I-b2microglobulin complex by the peptide-loading complex (tapasin, calreticulin, Erp57). The peptide-MHC-I complexes are translocated to the cell surface, where they are recognized by CD8^+^ T cells. In parallel, cDC1 dendritic cells cross-present tumor antigens to prime naïve T cells in the lymph node. Finally, at the tumor site, cytotoxic engagement occurs, leading to the targeted elimination of cancer cells. Quantitative or qualitative defects at any step (antigen processing, translocation, or MHC assembly) reduce tumor immunogenicity and contribute to immune evasion. Tumors often downregulate, rather than completely lose, HLA-I to avoid NK cell-mediated “missing-self” lysis [80,81,82]. 

In MSI-H/dMMR CRC, the high TMB increases the availability of immunogenic neoantigens, but effective presentation remains a prerequisite. The APM acts as a gatekeeper of which epitopes enter the immunopeptidome, thereby influencing the response to ICI and antigen-targeted strategies. Genomic studies have demonstrated that immune-escape drivers (HLA loss of heterozygosity, *B2M*, and *TAP* mutations) are frequent even in hypermutated tumors, underscoring the strong immune pressure to evade antigen presentation [83,84].

Clinically, the NICHE-2 trial demonstrated that pathological complete response (pCR) rates were high irrespective of *KRAS*/*BRAF* status. The rates were also observed in *B2M* mutant tumors, suggesting that APM defects may be partial or spatial and can be overcome in highly inflamed early-stage disease [7]. Spatial transcriptomic analyses revealed that an “interferon-high” immunophenotype, enriched in cytotoxic CD8^+^ T cells and antigen-presenting macrophages expressing CD74/MHC-II. This correlates with the benefit from PD-1 blockade. While this phenotype is common in dMMR CRCs, it is not exclusive and has been identified in a subset of pMMR CRC patients who may respond despite low TMB [85]. Single-cell RNA-seq studies have demonstrated that, compared to responders, anti-PD-1 resistance in dMMR CRC patients is often associated with IL-1b-driven MDSCs infiltration and CD8^+^ T-cell dysfunction, suggesting IL-1b is also a potential target [86].

Pharmacological modulation of the APM is emerging as a rational strategy. Preclinical data indicated that ATM inhibition activates cGAS/STING and NF-κB/IRF1/NLRC5 pathways, upregulates MHC-I, increases CD8^+^ T-cell infiltration, and sensitizes tumors to radiotherapy plus PD-1 blockade in CRC cells [87]. Conversely, *CEMIP*, an oncogene expressed significantly higher in CRC tissue than in normal colonic mucosa and associated with a malignant phenotype and poor clinical prognosis, promotes clathrin-mediated internalization and lysosomal degradation of MHC-I, impairing cytotoxic activity, while *CEMIP* inhibition synergizes with ICI in CRC models [88]. In addition, spatial multiomics highlight that MHC-II^+^ C1QC^+^ macrophages co-localize with CD4^+^ T cells in responders (irrespective of microsatellite status), whereas cancer-associated fibroblasts impair this interaction in non-responders [89]. Finally, targeting inhibitory myeloid checkpoints such as SIRPa has been found to restore phagocytosis and enhance antigen presentation, amplifying T-cell priming [90].

Comprehensively, an intact APM is a prerequisite, but insufficient in isolation, for ICI efficacy. Genetic, epigenetic, and pharmacological strategies aimed at restoring or enhancing APM functions represent potential strategies to convert “cold” CRCs into ICI-responsive tumors.

### 4.2. PD(L)1

Effective PD-1/PD-L1 engagement presupposes antigen visibility. Hence, APM integrity sets the stage for T-cell priming, as well as subsequent costimulatory circuits, including PD(L)1-govern expansion, function, and exhaustion of tumor-reactive T cells. T-cell-mediated tumor control requires efficient priming by cDC1, TCR engagement (signal 1), co-stimulation (signal-2), and cytokine support (signal 3). In CRC, innate-sensing pathways (e.g., TLRs, RIG-I, cGAS/STING) influence dendritic cell maturation, CXCL9/10 production, and CD8^+^ recruitment, thereby conditioning the amplitude of PD-1/PD-L1 interactions during effector phases [10].

Clinically, PD-L1 assessment is reported as tumor-cell proportion score (TPS) or combined positive score including immune cells (CPS). However, in colorectal cancer—particularly in MSI-high tumors—PD-L1 expression on immune cells within the tumor microenvironment may carry greater prognostic and biological relevance than tumor-cell expression alone. In this context, PD-L1 positivity often reflects an inflamed immune microenvironment rather than intrinsic tumor-driven immune evasion.

Moreover, spatial heterogeneity at the tumor-immune interface further complicates interpretation, as PD-L1 expression may vary between the invasive margin and the tumor core. To improve reproducibility and cross-study comparability, future studies should adopt standardized reporting frameworks that clearly distinguish between tumor-cell PD-L1 expression (TPS), combined scoring systems (CPS), and immune-predominant assessments (e.g., immune cell proportion score or IPS). Such compartment-specific reporting may better capture the complex immunobiology of colorectal cancer and refine its predictive and prognostic utility.

Importantly, the value of PD-L1 expression in CRC is strongly dependent on disease setting and cellular compartment. In the metastatic MSI-H/dMMR setting, PD-L1 expression has not demonstrated consistent predictive value and is not required for patient selection. However, emerging signals in locally advanced and neoadjuvant contexts suggest a potential role for PD-L1–mediated immune activation in modulating treatment response. In a randomized phase II trial in pMMR locally advanced rectal cancer, the addition of sintilimab (anti-PD-1 antibody) to neoadjuvant chemoradiotherapy significantly increased complete response rates, with more prominent benefits in CPS-positive tumors [91]. Emerging data also suggest that PD-L1 biology in CRC is compartment-dependent. Among resected CRCs, PD-L1 expression on immune cells, rather than tumor cells, was found to be associated with better prognosis, underscoring assay, and threshold variability [92].

Mechanistically, PD-L1 stability and surface expression are regulated by CKLF-like MARVEL transmembrane domain-containing protein 6 (CMTM6), which prevents PD-L1 degradation. In CRC patient cohorts, high stromal CMTM6 expression correlates with increased PD-L1 levels, higher TILs, and better prognosis, substantiating a biology of “inflamed but restrained” tumors [93]. Moreover, PD-L1 expression is dynamically modulated under therapeutic pressure. In MSS mCRC, treatment with cetuximab plus avelumab led to the selection of PD-L1 mutant subclones under Fc-competent pressure while simultaneously enhancing T-cell cytotoxicity, underscoring the dynamic and context-dependent nature of this biomarker [94].

Collectively, PD-1/PD-L1 assessment, preferably through CPS and with attention to cellular compartment, may enrich for benefit in select CRC settings, particularly in combination strategies. However, its predictive value remains unproven or incompletely investigated in pivotal metastatic ICI trials. Standardized assays, compartment-aware reporting, and integration with T-cell activation and innate immune activation signatures are essential to define its role across disease settings.

Collectively, these observations indicate that PD-L1 expression in CRC functions primarily as a context-dependent marker of immune engagement rather than a standalone predictive biomarker, with its relevance increasingly evident in early disease settings and immune-rich microenvironments.

#### 4.2.1. Microbiome

The gut microbiome is a newly recognized gatekeeper of antitumor immunity and, consequently, ICI efficacy. A complex network of molecular patterns, metabolites, and immune signaling pathways is emerging. Recent reviews demonstrate that in CRC, the microbiome is often characterized by dysbiosis, with an enrichment of “oral” pathobionts (e.g., *Fusobacterium*, *Parvimonas*, *Peptostreptococcus*) and a depletion of short-chain fatty acid (SCFA)-producing commensals, which collectively influence antigen presentation, myeloid polarization, and T-cell function. Importantly, intratumoral microbes can persist across disease sites and shape local immune tone, acting as biomarkers of response or resistance to ICIs, with context-dependent and sometimes paradoxical effects [95].

Among the most significant findings, *Fusobacterium nucleatum*, classically regarded as a prototypical immune-escape bacterium, has demonstrated a paradoxical dual role. In MSS CRC, high intratumoral Fn or fecal microbiota transplantation (FMT) from Fn-high donors unexpectedly sensitized tumors to PD-1 blockade through a butyrate-HDAC3/8-T-bet axis that alleviates CD8^+^ T-cell exhaustion [96]. Conversely, other studies demonstrated that Fn-derived succinic acid can suppress cGAS-IFNβ signaling and hinder CD8^+^ cell trafficking, driving immune resistance [97]. These apparently opposite effects highlight how microbial function (metabolic output and niche interactions) may provide a more complex biomarker than mere microbial presence. *Peptostreptococcus anaerobius* stimulates a2b1-integrin-CXCL1-CXCR2^+^ MDSC recruitment and immune exclusion, while pharmacological blockade of integrin or CXCR2 restores sensitivity to PD-1 inhibition [98].

On the other hand, several commensal species emerge as consistent “immune enhancers.” *Lactobacillus gallinarum* produces indole-3-carboxylic acid (ICA), which inhibits IDO1/kynurenine/AHR pathway, reduces intratumoral Tregs, and synergizes with PD-1 blockade [99]. In contrast, microbiota-derived deoxycholic acid (DCA) acts as a metabolic brake, mitigating CD8^+^ effector function through PMCA-NFAT inhibition, linking bile acid metabolism to resistance phenotypes [100]. *Roseburia intestinalis*, a butyrate-producing commensal, is significantly depleted in patients with CRC compared to healthy controls. In murine models, restoring this microbe demonstrated suppressed tumor growth and enhanced anti-PD-1 efficacy in both MSI-high and low CRCs, inducing granzyme B^+^, IFN-g, TNF-a, and CD8^+^ T cells infiltration [101]. *Clostridium butyricum* has demonstrated potential as an immune-sensitizing bacterium in both MSS and MSI-H CRCs. Single-cell transcriptomic analyses indicated that bacterial surface protein mediates GRP78 inhibition and PI3K-AKT-NF-kB down streaming, reducing IL-6 secretion, mitigating myeloid-driven T-cell suppression, and enhancing T-lymphocyte activation [102].

Clinically, a phase II trial combining FMT, anti-PD-1, and anti-VEGFR therapy in refractory MSS mCRC demonstrated durable disease control and manageable safety [103]. Spatial transcriptomics revealed that intratumoral bacteria are primarily within myeloid cells, which serve as microbe-rich hubs. Infected tumor cells upregulated APM, including MHC-related pathways, suggesting a direct connection between intratumoral microbiota and adaptive immunity [104].

Collectively, these data present the microbiome as a dynamic and potential immune biomarker to refine patient stratification and provide a rationale for therapeutic combination strategies. Nevertheless, several limitations must be acknowledged. The microbiome represents a highly heterogeneous and context-dependent biomarker, with significant biological differences between fecal and intratumoral microbial communities, which may convey distinct and sometimes non-overlapping immunological signals. Moreover, substantial inter-study variability, discrepancies in sampling methods, sequencing platforms, analytical pipelines, and patient populations currently limit reproducibility and cross-trial comparability. Consequently, microbiome modulation in CRC should presently be regarded as a hypothesis-generating and exploratory strategy rather than a practice-changing approach, requiring standardized methodologies and prospective validation before clinical implementation.

#### 4.2.2. Circulating Tumor DNA

In the immunotherapy era, circulating tumor DNA (ctDNA) is emerging as a minimally invasive biomarker capable of identifying tumor heterogeneity and monitoring of dynamic evolution during treatment [105,106]. In CRC, baseline ctDNA levels correlate with tumor burden and prognosis, while dynamic fluctuations during therapy provide diagnostic anticipation of disease trajectory [107,108].

In patients with MSI-H CRC, where identifying primary resistance and defining safe discontinuation criteria for durable responders remain key challenges, ctDNA monitoring represents a valuable adjunct tool, although its role is yet to be established. Notably, none of the pivotal trials in MSI-H mCRC (KEYNOTE-177, CheckMate-8HW, NICHE-2) have incorporated ctDNA analyses, leaving its clinical utility in this context largely unexplored.

Recent studies highlight the first evidence of prognostic and predictive relevance of ctDNA kinetics under ICI therapy. In the secondary analysis of the SAMCO-PRODIGE 54 trial, early ctDNA reduction one month after treatment initiation predicted both PFS and OS in dMMR/MSI-H mCRC, particularly among patients receiving avelumab [109]. Similar findings were also observed in the prospective ADI-MSI study: patients exhibiting dMMR/MSI-H CRCs with decreasing or undetectable ctDNA-MSI levels experienced superior long-term outcomes compared to those with increasing levels. ctDNA-MSI kinetics remained an independent prognostic factor in multivariate analysis [110]. A meta-analysis of 18 ICI trials across solid tumors confirmed that ctDNA decline, typically >50% or to undetectable levels, correlates with improved outcomes (HR ≈ 0.2 for PFS and OS) [111]. Phase II data demonstrate similar trends: MSI-H CRC patients with ctDNA clearance following neoadjuvant pembrolizumab had a 3-year event-free survival (EFS) of 92% compared to 20% for ctDNA-positive counterparts [112], and baseline ctDNA negativity or on-treatment clearance predicted pCR with an 80% probability [113]. Meta-analytic evidence supports this association while underscoring inter-assay heterogeneity and CRC representation [114].

From a translational perspective, ctDNA persistence indicates molecular residual disease and early resistance [115,116], while its clearance is associated with radiographic and immunologic responses across mCRC subtypes [117]. Conversely, early ctDNA rise often precedes radiological progression, even in MSS under ICI combination, suggesting a role for real-time treatment adaptation [118].

Overall, ctDNA represents a potential biomarker complementing radiologic and clinical evaluation, enabling personalized and dynamic monitoring of response, resistance, and long-term benefit in MSI-H and selected MSS CRC. An important unresolved question is whether ctDNA kinetics, integrated with radiological assessment, could inform real-time adaptation of ICI therapy. Persistent or rising ctDNA levels despite radiological stability may reflect molecular resistance preceding RECIST-defined progression, potentially identifying patients who could benefit from early treatment intensification or combination strategies. Conversely, sustained ctDNA clearance in deep responders raises the possibility of treatment de-escalation or duration tailoring. However, such ctDNA-guided treatment modification remains investigational, as prospective interventional trials validating adaptive strategies are currently lacking. Additionally, technical heterogeneity among assays and the absence of standardized cutoffs remain key barriers to its routine clinical implementation.

#### 4.2.3. MicroRNAs and Post-Transcriptional Regulation of Immunotherapy Response

Beyond genomic and microenvironmental determinants, microRNAs (miRNAs) have emerged as important post-transcriptional regulators of the immune response in colorectal cancer. miRNAs can modulate multiple components of the cancer–immunity cycle, including antigen presentation, interferon signaling, T-cell activation, and immune checkpoint expression. Several studies suggest that dysregulated miRNA networks may partially explain the discordance between MMR/MSI status and clinical response to immune checkpoint inhibitors. For example, specific miRNAs have been shown to regulate PD-L1 expression directly or indirectly, influencing immune-escape mechanisms independently of tumor mutational burden. Others modulate key inflammatory mediators, such as STAT1/STAT3 and interferon-γ signaling pathways, thereby shaping the immune contexture of the tumor microenvironment. In this regard, miRNA-driven immune modulation may act as a confounding factor in predicting ICI responsiveness, even among tumors classified as MSI-H/dMMR [119]. These observations suggest that integrating miRNA profiling with established biomarkers could refine patient stratification and better explain heterogeneous responses within molecularly defined subgroups.

### 4.3. Conclusions and Future Directions

Immunotherapy has dramatically transformed the therapeutic landscape of CRC; however, its clinical benefit is still limited to a biologically selected subset of patients. Deficient mismatch repair and microsatellite instability represent the main biomarkers for safely selecting ICIs, predicting durable and sustained responses across disease stages. However, MSI status alone is insufficient to capture the heterogeneity of immunotherapy response. This review elucidates how ICI response or resistance results from the complex and dynamic interplay between tumor-intrinsic biology, microenvironment, proficient immune system activation, and systemic factors. Canonical biomarkers, including TMB or *POLE*/*POLD* mutations, predict hypermutated and potentially highly immunogenic tumors; however, their utility is not without outcome variability. Conversely, selected concomitant molecular alterations and microenvironmental composition may contribute to immune escape, secondary resistance, or incomplete responses, despite a favorable genomic background. Emerging evidence demonstrates the relevance of dynamic biomarkers, such as circulating DNA fluctuations, which may monitor minimal residual disease, early resistance, and track molecular response, potentially guiding therapeutic decisions. Similarly, microbiome-driven modulation of the immune niche suggests a potential therapeutic strategy. The future of immunotherapy necessitates prospective trials integrating longitudinal biomarkers, spatial and single-cell immune profiling, and combination strategies to extend ICI benefits beyond the MSI-H population, refine patient selection, and advance toward truly personalized immune-oncology in CRC.

## Figures and Tables

**Table 1:** Canonical biomarkers of response to immune checkpoint inhibitors in colorectal cancer.

Biomarker	Prevalence in CRC	Predictive Value for ICI	Clinical Setting
MSI-H/dMMR	~15% overall CRC; ~3–5% mCRC	Strongly predictive of response	Standard of care for ICIs across disease stages
TMB	Variable, enriched in MSI-H/dMMR and *POLE*/*POLD* tumors	Context-dependent	Not recommended as a standalone biomarker; higher cutoffs (≥50 mut/Mb) suggested
*POLE*/*POLD* pathogenic variants	<1% CRC	Highly predictive	Responses observed in MSS tumors as well
TME	100% (qualitative feature)	Modulatory/ complementary	High CD8+ TILs, TLS, IFN-γ signaling favorable responses, stromal and metabolic barriers confer resistance

Abbreviations: CD8^+^ TILs, CD8^+^ tumor-infiltrating lymphocytes; CRC, colorectal cancer; dMMR, deficient mismatch repair; ICIs, immune checkpoint inhibitors; mCRC, metastatic colorectal cancer; MSI-H, microsatellite instability-high; MSS, microsatellite stable; TLS, tertiary lymphoid structures; TMB, tumor mutational burden; TME, tumor microenvironment.

**Table 2:** Concomitant molecular alterations.

Biomarker	Prevalence in CRC	Predictive Value for ICI	Key Note
*BRAF* V600E	~8–12%, enriched in MSI-H	Context-dependent	Secondary resistance Benefit from dual ICI
*KRAS*	~40%, mostly MSS	Neutral in MSI-H	Immunomodulatory role
*HER2*	~3–5% MSS, rare in MSI-H	Possible resistance	Immune-cold phenotype
*PI3K*/*PTEN*	~20–25%, variable association with MSI-H	Negative	Myeloid-driven escape
*NTRK* fusions	<1%, enriched in MSI-H	Potentially positive	Hypermutated subtype
*STK11*	Rare, mostly MSS	Potentially negative (extrapolated)	Limited CRC data

Abbreviations: CRC, colorectal cancer; MSI-H, microsatellite instability-high; MSS, microsatellite stable.

## Data Availability

No new data were created or analyzed in this study.
